# Prognostic parameters and detection of cardiac amyloidosis with hybrid ^18^F-Florbetaben-PET/MRI: an exploratory observational study

**DOI:** 10.1007/s00259-025-07733-x

**Published:** 2026-02-07

**Authors:** Lukas Kessler, Wibke Tonscheidt, Kai Nassenstein, Stephan Settelmeier, Alexander Carpinteiro, H. Christian Reinhardt, Tim Hagenacker, Lale Umutlu, Christoph Kleinschnitz, Simon Wernhart, Lars Michel, Michal K. Chodyla, Benedikt M. Schaarschmidt, Thomas-Wilfried Schlosser, Wolfgang P. Fendler, Francesco Barbato, Maria Papathanasiou, Christoph Rischpler, Ken Herrmann, Tienush Rassaf, David Kersting

**Affiliations:** 1 Present Address: Department of Nuclear Medicine, University Hospital Essen, Hufelandstrasse 55, 45147 Essen, Germany; 2 Present Address: Department of Diagnostic and Interventional Radiology and Neuroradiology, University Hospital Essen, Hufelandstrasse 55, 45147 Essen, Germany; 3 Department of Cardiology and Vascular Medicine, West German Heart and Vascular Center, University Hospital Essen, Hufelandstrasse 55, 45147 Essen, Germany; 4 Department of Hematology, West German Tumor Center, University Hospital Essen, Hufelandstrasse 55, 45147 Essen, Germany; 5 Department of Molecular Biology, University of Duisburg-Essen, Hufelandstrasse 55, 45147 Essen, Germany; 6 Department of Neurology, University Hospital Essen, Hufelandstrasse 55, 45147 Essen, Germany; 7 Present Address: Department of Preventive Sports Medicine and Sports Cardiology, TUM School of Medicine and Health, TUM University Hospital Klinikum rechts der Isar, Technical University of Munich, Georg-Brauchle-Ring 56, D-80992 Munich, Germany; 8 Present Address: Department of Department of Cardiology/Angiology, University Heart Center Frankfurt, Goethe University Hospital, Frankfurt, Germany; 9 Present Address: Department of Nuclear Medicine, Klinikum Stuttgart, Prießnitzweg 24, 70374 Stuttgart, Germany; 10 Present Address: West German Amyloidosis Center, Hufelandstrasse 55, 45147 Essen, Germany

**Keywords:** Cardiac amyloidosis, Positron emission tomography, Amyloid-binding tracers, Molecular imaging, Transthyretin, Light-chain

## Abstract

**Purpose:**

Positron emission tomography (PET) with amyloid-binding tracers was shown to have high sensitivity for the detection of both transthyretin (ATTR) and light-chain (AL) cardiac amyloidosis (CA). Recent studies describe prognostic value of imaging biomarkers from bone scintigraphy and ^18^F-Florbetapir. The aim of this study was to evaluate the value of imaging biomarkers from ^18^F-Florbetaben PET, cardiac magnetic resonance (CMR), and echocardiography imaging for prediction of major adverse cardiac events (MACE) in comparison to serum biomarkers in patients with different types of CA.

**Methods:**

Patients who underwent cardiac ^18^F-Florbetaben PET/MRI were prospectively enrolled and received clinical follow-up for up to 36 months and MACE were reported (NCT07154381). Scans were reported by two blinded, nuclear medicine physicians. Imaging biomarkers including average retention index (RI), T1 mapping/ extracellular volume (ECV) and serological markers were estimated and their association with MACE free survival was analyzed.

**Results:**

Twenty-one patients with confirmed CA were enrolled. MACEs were reported in 14 of 21 patients (66.7%). Higher average RI was the only imaging biomarker that was a significant predictor for MACE in uni- and multivariate analysis (HR = 4.02, 95%CI: 1.25-12.9, p < 0.05). N-terminal pro-B-type natriuretic peptide (NT-proBNP) was a significant predictor in uni- but not in multivariate analysis. Patients with AL-CA showed a higher rate of MACE than patients with other subtypes.

**Conclusion:**

Integrated ^18^F-Florbetaben PET/MR allows diagnosis, subtype differentiation and outcome predication of CA. The average RI was the only significant and independent prognostic imaging biomarker of MACE. Future prospective studies are warranted to investigate benefits for patient management and risk assessment in larger cohorts.

**Supplementary Information:**

The online version contains supplementary material available at 10.1007/s00259-025-07733-x.

## Introduction

Cardiac amyloidosis (CA) is as a significant cause for progressive heart failure (HF), marked by elevated mortality rates [1]. Previously considered rare, CA was predominantly linked to immunoglobulin light-chain derived amyloid (AL), associated with plasma cell dyscrasia. Transthyretin amyloidosis (ATTR) has emerged as highly prevalent among older patients exhibiting left ventricular hypertrophy, HF with preserved ejection fraction (HFpEF), and conduction disorders [2–5]. Despite increased disease recognition, it is a relatively underdiagnosed heart disease. ATTR-CA and AL-CA constitute the majority of CA cases, both carrying a grim prognosis. In AL-CA, median survival can be less than 6 months from diagnosis, contingent on the severity of cardiac involvement [6]. In ATTR amyloidosis, the acquired, wild-type disease has a more extended course, while the hereditary forms’ prognosis varies based on the underlying transthyretin gene mutation [7].

Advancing cardiac imaging techniques is deemed are crucial for an earlier disease detection and diagnosis. Cardiac magnetic resonance imaging (CMR) enables tissue characterization and facilitates differential diagnosis of myocardial hypertrophy, but specificity and CA-subtype differentiation by CMR is limited [8]. In contrast, bone-tracer scintigraphy has become a cornerstone as a non-invasive diagnostic tool for ATTR-CA with high diagnostic accuracy. Despite this paradigm shift, bone-tracer scintigraphy does not enable non-invasive diagnosis of AL-CA and scarce data exist regarding its diagnostic yield at an early disease stage. Targeted molecular imaging of cardiac amyloid deposits using ^18^F- and ^11^C-labelled radiotracers has been shown feasible for both AL- and ATTR-CA in the last years. A variety of studies investigated the accuracy of positron emission tomography (PET) imaging using ^18^F-labelled Florbetapir, ^18^F-Florbetaben and Flutemetamol, as well as ^11^C-Pittsburgh compound B for the detection of amyloid in the heart [9–16]. Notably, AL patients exhibited a higher myocardial tracer activity than ATTR patients, indicating a different binding mechanism and distinct biologic properties of ATTR- and AL-derived amyloid. Although these studies typically involved small sample sizes, a meta-analysis of six studies with 98 patients reported a sensitivity of 95% and a specificity of 98% for these tracers [17–19] and analysis of kinetic PET parameters allows to discriminate between AL- and ATTR-CA [10–12].

Next to these diagnostic qualities, a recent study describes the potential of imaging biomarkers derived from ^18^F-Florbetapir PET to predict major cardiac adverse events (MACE) in patients with AL-CA [20]. However, no systematic evaluation in patients with different CA subtypes was performed so far. Prognostic parameters for CMR have been thoroughly described in recent years and show that fibrosis/scarring (measured by T1 mapping/ECV and LGE) is a key prognostic factor for various cardiac diseases including cardiac amyloidosis [21]. Nonetheless these parameters are not disease specific for cardiac amyloidosis and vary between subtypes. The aim of this prospective observational trial was to investigate the value of imaging biomarkers focused on ^18^F-Florbetaben PET in context to cardiac magnetic resonance (CMR), echocardiography imaging and comparison to serum biomarkers for prediction of MACE in patients with ATTR-, AL- and AA-CA.

## Methods

### Study patients and study design

We prospectively enrolled patients with proven cardiac amyloidosis who underwent ^18^F-Florbetaben PET/MRI as part of clinical diagnostic work-up in this observational trial (NCT07154381). CA was diagnosed based on the criteria defined in current practice guidelines [1, 22]: All study participants underwent a baseline diagnostic evaluation including medical history, physical examination, 12-lead electrocardiogram, transthoracic echocardiogram, and laboratory tests for plasma cell proliferative disease (serum free light chain assay, serum and urine immunofixation, and serum protein electrophoresis) according to our institution’s standardized procedures.

CA Patients were eligible for inclusion, if simultaneous cardiac PET/MRI in an integrated system with 3 Tesla field strength for measurement of amyloid burden was performed up in the diagnostic work up with uniform protocol (see below). Clinical follow up visits were evaluated at 6- and 12-months after PET/MRI imaging. Additionally major adverse cardiac events were reported for up to 36 months after PET/MRI imaging. The study was approved by the local institutional review board (University Duisburg-Essen registry number: 19–8798-BO). Informed consent was obtained from all individual participants included in the study, and all study procedures were in accordance with the institutional ethical standards and the declaration of Helsinki.

### Major cardiac adverse events

The following MACEs were considered for the time-to-first-event analysis: all-cause death, hospitalization due to heart failure, sustained ventricular tachyarrhythmia (≥ 30 s), implantation of a cardioverter defibrillator or other urgent cardiovascular interventions. MACE-free survival was calculated as the interval from PET imaging until onset of MACE.

### PET protocol and image analysis

Scans were performed on an integrated whole-body PET/MRI system (Biograph mMR, Siemens Healthcare, Erlangen, Germany). A mean activity of 230.4 ± 68.7 MBq of ^18^F-Florbetaben (Life Molecular Imaging, Berlin, Germany) was administered intravenously. All PET/MRI scans were acquired in PET list-mode acquisition for 60 min, starting simultaneously with tracer injection. Static and dynamic reconstructions were performed as previously described (Supplemental Material) [11, 12, 23]. CMR scans were performed using a clinical routine protocol including Cine, LGE and pre- and post-contrast T1 mapping sequences enabling ECV calculation, detailed protocol in Supplemental Material.

Left ventricular myocardial and blood pool standardized uptake values (SUV) were determined and normalized to bodyweight. Myocardialuptake parameters were measured by an automated 50% Iso-contur volume of Interest (VOI) in reconstructed list-mode data set (Syngo.via, Siemens Healthineers, Germany) and uptake per frame was automatically measured. Scans were visually inspected by two blinded, experienced nuclear medicine physicians and then reported as positive if myocardial tracer uptake was visually higher than the background uptake. Established retention metrics, namely myocardial retention index (RI), myocardial tracer retention (MTR) and tracer washout were calculated as previously described (definitions are given in Supplemental Material) [11, 12, 23].

### MRI protocol and image analysis

Cardiac MRI was performed simultaneously with PET on an integrated whole-body 3 Tesla PET/MRI system (Biograph mMR, Siemens Healthineers, Erlangen, Germany). Pre- and post-contrast T1 mapping as well as LGE study were performed as previously described [15].

### Statistical analysis

Continuous variables are summarized as means (standard deviations), unless indicated otherwise, and categorical variables as counts (percentages). Continuous data were evaluated for normality of distribution with the Shapiro–Wilk test. Kruskal-Wallis test was used for comparison of continuous parameters between CA subtypes. The x^2^ test or the Fisher’s exact test was used for testing the association between two categorical variables. MACE-free survival was analyzed by uni- and multivariate Cox regression analyses and visualized using Kaplan-Meier curves and Forest plots. Parameters were stratified by median. Hazard ratios (HR) with 95%-confidence intervals were calculated. Mediation analysis was performed after log transformation of variables in accordance to Clerk et al. [20]. Correlation analyses were performed using the Pearson correlation coefficient. For all analyses, P values < 0.05 were regarded as statistically significant. All analyses were performed using Graphpad Prism (Version 10.2.3 for Windows, GraphPad Software, Boston, Massachusetts USA) and R statistical software (Version 4.4.1. R Foundation for Statistical Computing, Vienna, Austria). See Supplemental Material for detailed statistics section.

## Results

### Patient cohort

We included 21 patients who underwent cardiac ^18^F-Florbetaben PET/MRI imaging from 03/2020 to 08/2021. Among these, 12 patients were diagnosed with wild-type ATTR-CA (wtATTR), 3 with hereditary ATTR-CA (hATTR), 4 with AL-CA (AL), and 2 with Serum-Amyloid A (AA) -CA. The baseline characteristics of patients are shown in Table 1.

**Table 1:** Baseline characteristics

	Mean ± SD or No. of patients (%)
Age (y)	70.1 ± 11.4
Gender (m/f)	14/7 (67/33%)
BMI (kg/m^2^)	26.8 ± 4.3
**Amyloid Type**	
wtATTR	12 (57.1)
hATTR	3 (14.3)
AL	4 (19.1)
AA	2 (9.5)
**NYHA Class**	
0	1 (4.8)
I	2 (9.5)
II	12 (57.1)
III	6 (28.6)
NT-proBNP (pg/ml) *	10,033 (Range 164–64397)
eGFR (ml/min/1.73 m^2^)	60.0 ± 11.0
**Heart Failure**	
No HF	4 (19.0)
HFpEF	10 (47.6)
HFmrEF	3 (14.3)
HFrEF	4 (19.1)

*AL* Light-Chain Amyloid, (*wt*/*h*)*ATTR* (wild-type/hereditary) Transthyretin Amyloid, *BMI* Body-Mass Index, *eGFR* Estimated Glomerular Filtration Rate, *NT*-*proBNP* N-Terminal-pro B-Type Natriuretic Peptide, *NYHA* New York Heart Association. * The given values represent median (min-max). HF(p, mr, r,)EF = heart failure with preserved, mildly reduced or reduced ejection fraction

**Fig. 1:**
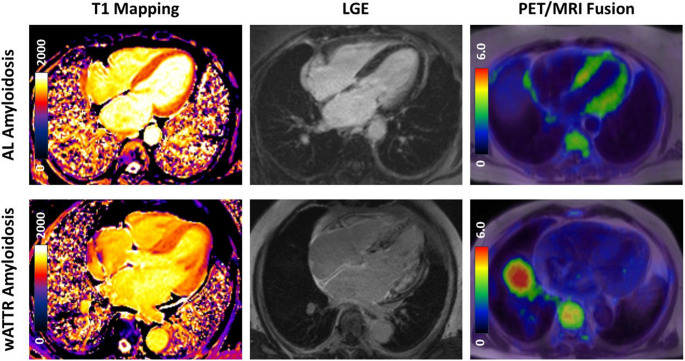
Representative case examples. (A) ^18^F-Florbetaben PET/MRI of a PET-positive, female patient with AL-amyloidosis. CMR showed abnormal mean T1-Relaxation in native T1 Mapping (1319 ms) and mild LGE, with increased ^18^F-Florbetaben uptake (SUVmax 5.6) 40–60 min post-injection. (B) negative PET of wtATTR male patient with abnormal T1 relaxation (1360 ms), strong LGE of left and right ventricular myocardium with only faint tracer uptake (SUVmax 3.3)

Figure 1 demonstrates representative patient examples. First, a patient with AL-CA showing strong cardiac uptake PET and with weak LGE of the left ventricular myocardium is presented; average RI was 0.26. The patient was hospitalized after 22 months of follow-up due to decompensated heart failure. Second, a patient with wtATTR-CA without cardiac PET uptake and diffuse LGE is shown; average RI was 0.03, no MACE occurred during follow-up.

### Differentiation of CA subtypes

Analysis of imaging parameters showed several differences between subtypes of CA (Table 2). Especially PET parameters provided good discrimination between AL-CA and wtATTR subtype with overall higher uptake values (for example, AL-CA SUVmax 6.6 ± 1.2 vs. wtATTR-CA 2.7 ± 1.0; *p* = 0.007) and significant differences in all kinetic parameters (for example, RI 15 to 20 min AL-CA 0.15 ± 0.05 vs. wtATTR-CA 0.05 ± 0.02; *p* = 0.006).

**Table 2:** Imaging data

	Amyloidosis *n* = 21	wATTR *n* = 12	hATTR *n* = 3	AL *n* = 4	AA *n* = 2	*P*-Value
**MACE**,** No. (%)**	14 (66.7%)	7 (58.3%)	1 (33.3%)	4 (100%)	2 (100%)	0.23
**Echocardiography**						
LVEF (%)	49.5 ± 10.1	45.1 ± 13.0	58.4 ± 6.6	50.9 ± 6.3	26.0 ± 11.4	0.06
IVSd (mm)	20.5 ± 4.6	16.8 ± 4.1	22.3 ± 10.1	20.0 ± 7.3	12.5 ± 3.5	0.30
LAVI (g/m^2^)	43.8 ± 15.3	46.6 ± 10.3	45.3 ± 7.1	32.5 ± 26.0	50.5 ± 20.5	0.72
LV-GLS	−10.4 ± 5.4	−10.7 ± 5.4	−15.5 ± 1.2	−9.1 ± 4.2	−4.1 ± 5.9	0.16
E/E’	14.4 ± 7.3	10.4 ± 2.1	11.8 ± 5.2	21.2 ± 8.6	25.0 ± 7.1	**0.002**
**PET**						
SUV max	3.5 ± 1.9	2.7 ± 1.0	3.1 ± 1.0	6.6 ± 1.2	2.3 ± 0.4	**0.007**
SUV mean	2.0 ± 1.2	1.4 ± 0.5	1.6 ± 0.4	4.1 ± 1.0	1.4 ± 0.2	**0.004**
SUV peak	2.9 ± 1.7	2.3 ± 0.6	2.4 ± 0.8	5.6 ± 1.2	2.1 ± 0.4	0.11
Retention Index	0.07 ± 0.05	0.05 ± 0.02	0.05 ± 0.02	0.15 ± 0.05	0.04 ± 0.01	**0.006**
Washout	50.5 ± 18.4	57.8 ± 8.5	62.1 ± 11.9	19.1 ± 14.3	52.3 ± 8.5	**0.002**
MTR	46.6 ± 37.8	33.0 ± 14.8	29.9 ± 16.8	110.5 ± 42.0	25.5 ± 3.0	**0.007**
**CMR**						
LVEF (%)	46.4 ± 13.4	45.1 ± 13.0	58.4 ± 6.6	50.9 ± 6.2	*26.0* ± 11.4	0.06
LVMM index (g/m^2^)	81.1 ± 25.0	86.1 ± 30.1	72.1 ± 18.0	*75.4* ± 24.1	81.5 ± 6.0	0.91
T1 Relaxation Time (ms)	1348 ± 82.3	1327 ± 88.7	1402 ± 33.7	1393 ± 77.2	1285 ± 40.3	0.12
ECV (%)	45.5 ± 9.8	45.5 ± 10.4	46.7 ± 8.4	48.5 ± 11.1	35.0 ± 0.0*	0.83
Late Gadolinium Enhancement	18 (85.7)	10 (83.3%)	3 (100%)	4 (100%)	1 (50%)	

*E*’ Peak Velocity of Early Diastolic Mitral Annular Motion, *E*/*E*’ Ratio of Peak Velocity of Early Diastolic Transmitral Flow to Peak Velocity of Early Diastolic Mitral Annular Motion, *IVSd* End-diastolic Diameter of the Intraventricular Septum, *LAVI* Left Atrial Volume Index (g/m2), *LVEF* Left Ventricular Ejection Fraction, *LVMM* Left Ventricular Muscle Mass, *GLS* global longitudinal strain, *SUV* Standardized Uptake Value at 40–60 min p.i. *MTR* myocardial tracer retention, *RI* retention index, *ECV* Extracellular Volume

For echocardiographic parameters, only E/E’ showed significant differences for amyloidosis subtypes (*p* = 0.002) with higher values for AL-amyloidosis. CMR parameters did not show significant differences between CA subtypes. Details are given in Table 2.

### Univariate MACE-free survival analysis

The median follow-up time was 33.3 months. Most frequent causes of hospitalization were heart failure with acute decompensation (64.3%), followed by myocardial infarction/coronary artery disease with necessary intervention (21.4%). One patient died in the follow up period (7.1%) and one patient underwent ICD implantation (7.1%) (details in Supplemental Table 1).

In univariate analysis (Table 3), the PET parameter average RI (HR = 4.02, 95%CI: 1.25–12.9, *p* = 0.019) and the serum parameters creatinine (HR = 14.1, 95%CI: 2.90–68.6, *p* = 0.001), hsTrop (HR = 5.41, 95%CI: 1.60–18.3, *p* = 0.007), and NT-proBNP (HR = 4.85, 95%CI: 1.45–16.2, *p* = 0.010) were significantly associated with shorter MACE free survival. For the VCI diameter statistical significance was just not reached but with a trend towards significance (HR = 3.31, 95%CI: 1.00–11.0, *p* = 0.051). The other investigated parameters did not show significant association with MACE free survival (Table 3).

**Table 3:** Univariate regression analysis

	Risk Factor	Stratification	HR	95% CI	Median MFS (d) (low risk vs. high risk)	*p*
**Demographics**	Age	> 72 y	0.96	0.33, 2.77	578 vs. 675	> 0.9
	Gender	male	1.67	0.55, 5.06	974 vs. 278	0.4
	BMI	> 26.7	0.60	0.21, 1.74	480 vs. 853	0.3
	Amyloid type	Al vs. no AL	2.05	0.63, 6.71	452 vs. 627	0.2
	NYHA	> 2	1.76	0.54, 5.76	673 vs. 231	0.3
**PET**	Visual Impression	“positive”	1.08	0.36, 3.24	578 vs. 673	0.9
	SUVmax	> 2.7	1.01	0.34, 3.01	381 vs. 673	> 0.9
	TBRpeak	> 0.054	1.59	0.53, 4.76	675 vs.578	0.4
	SUVmean	> 1.4	0.99	0.35, 2.85	497 vs.626	> 0.9
	MTR	> 29	1.08	0.37, 3.13	578 vs.673	0.9
	Washout	≤ 55.16	1.71	0.57, 5.13	675 vs.318	0.3
	Retention Index 15 to 20 min	> 0.05	2.13	0.3, 6.23	231 vs. 675	0.2
	Average Retention Index	> 0.16	4.02	1.25, 12.9	1,033 vs. 278	**0.019**
**CMR**	LVEF	≤ 50.85	0.77	0.27, 2.25	626 vs. 678	0.6
	T1 Relaxation time	> 1360	0.98	0.34, 2.80	527 vs. 627	> 0.9
	ECV	> 49	0.96	0.32, 2.88	496 vs. 675	> 0.9
**ECHO**	GLS	> −8.9	2.03	0.68, 6.09	675 vs. 318	0.2
	E/E` ratio	> 12.5	1.85	0.63, 5.37	1,033 vs. 381	0.3
	LVMMI	> 189	0.87	0.28, 2.71	381 vs. 675	0.8
	IVSD	> 16.5	0.83	0.28, 2.47	350 vs. 675	0.7
	VCI diameter	> 18	3.31	1.00, 11.0	1,033 vs. 298	0.051
**SERUM**	Creatinine	> 1.24	14.1	2.90, 68.6	1,033 vs. 229	**0.001**
	eGFR	≤ 60	1.24	0.40, 3.79	824 vs. 480	0.7
	LDH	> 229	3.04	0.93, 9.99	1,033 vs. 330	0.067
	hsTrop	> 46	5.41	1.60, 18.3	1,033 vs. 231	**0.007**
	NT-proBNP	> 2863	4.85	1.45, 16.2	1,033 vs. 318	**0.010**

*MTR* myocardial tracer retention, *ECV* extracellular volume, *GLS* LV global logintudinal strain. Numerical data were stratified by median

**Fig. 2:**
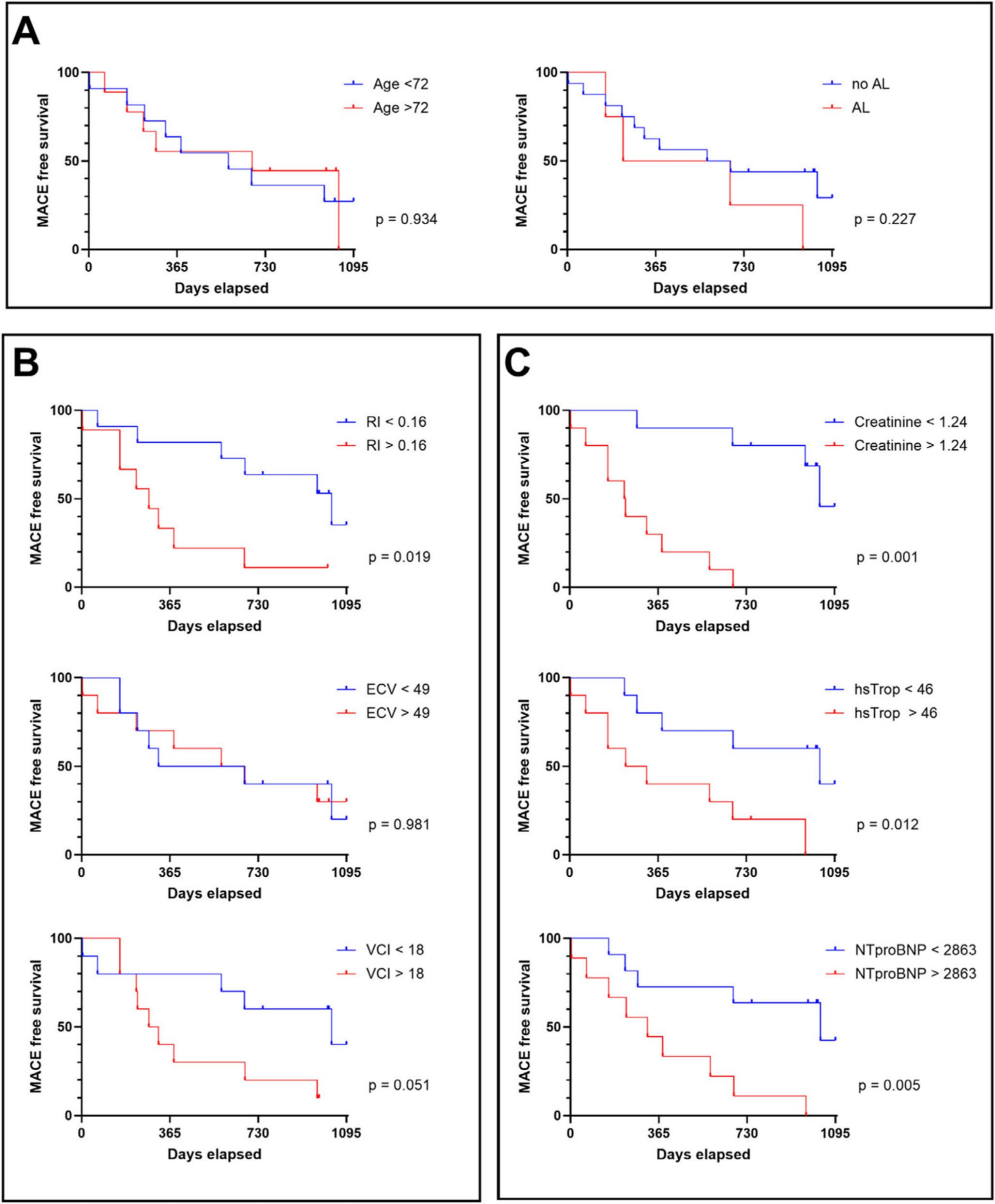
Kaplan-Meier-Curves of MACE free survival for selected parameters based on univariate analysis. (A) Age and AL vs. no AL amyloidosis subtype. (B) Imaging parameters. (C) serum biomarkers

Kaplan-Meier analyses for the parameters showing significant association and for other parameters with previously described predictive value in CA are shown in Fig. 2.

### Multivariate MACE-free survival analysis

The parameters from each diagnostic group (PET, MRI, echocardiography, serum) that showed significant or best association with MACE free survival and play a role in diagnosing cardiac amyloidosis (RI, ECV, VCI diameter, NT-proBNP, and creatinine) were analyzed in multivariate Cox regression. Amyloid type and age were included as these parameters may influence survival in patients with CA and are, therefore, relevant potential confounders.

We used AL vs. other subtype as a confounder due to fact that AL amyloidosis generally has worse survival compared to ATTR and AA-amyloidosis.

**Fig. 3:**
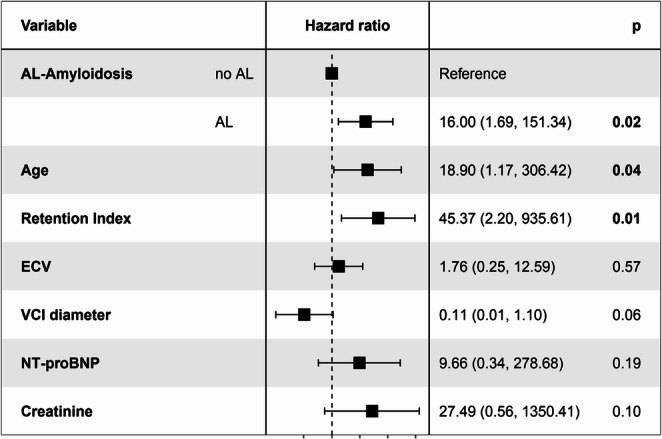
Forest plot of Hazard ratios by multivariate Cox-regression

In multivariate analysis, higher average RI still showed significant association with shorter MACE free survival (HR = 45.37, 95%CI: 2.20–935.61.20.61, *p* < 0.05). Moreover, AL-CA (HR = 16.00, 95%CI: 1.69–151.34.69.34, *p* < 0.05) and higher age (HR = 19.90, 95%CI: 1.17–306.42.17.42, *p* < 0.05) were significantly associated with shorter MACE free survival. A Forest plot showing detailed results is presented in Fig. 3.

### Mediation analysis

Next, we performed a mediation analysis to evaluate an association between left ventricular amyloid burden (expressed by average RI) and MACE through an indirect pathway via NT-proBNP (as a marker of heart failure) or via troponin T (as a marker of myocardial damage), since these associations were previously suggested for left ventricular ^18^F-Florbetapir uptake in patients with cardiac AL amyloidosis [20]. We observed no association between RI and NT-proBNP (*p* = 0.93) or RI and Troponin C (*p* = 0.31), indicating no mediation effect in our patient cohort.

## Correlation of imaging parameters

**Fig. 4:**
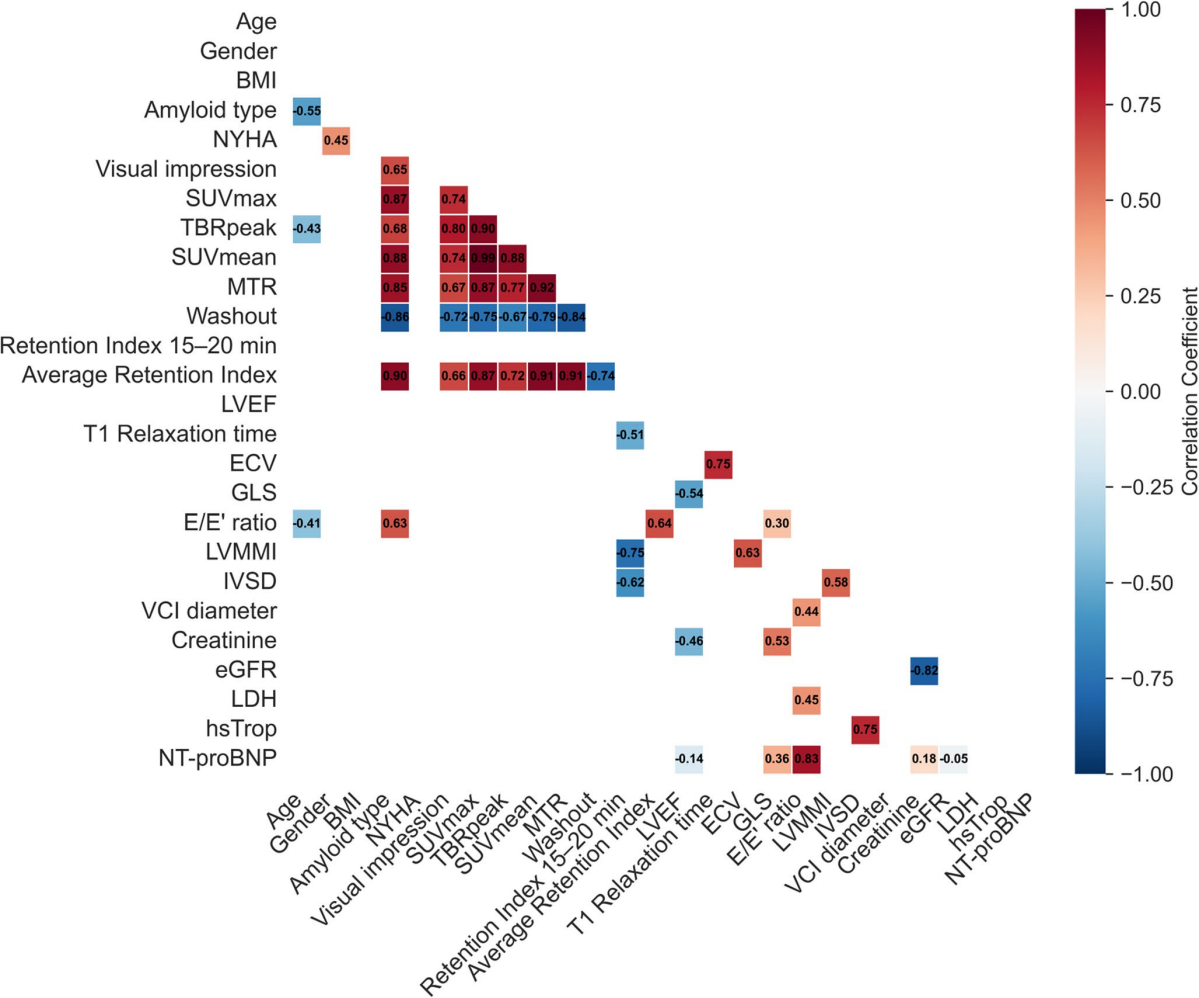
Correlation Matrix. Only significant correlations (p < 0.05) in colored boxes with respective Pearsons R (see color scale)

To understand possible interactions of imaging biomarkers from the different modalities, we performed a detailed correlation analysis using the Pearson correlation coefficient. Here, all PET parameters, including the RI from 15 to 20 min, showed strong positive relationships with each other and with AL-CA subtype except for washout parameter with strong negative relationship (explained by parameter calculation) and the average RI. Interestingly, the average RI, the only significant imaging biomarker to predict MACE free survival, showed moderate to strong negative correlations with the CMR parameter T1 Relaxation time (*r* = −0.60) and the echocardiography parameters left ventricular muscle mass index (LVMMi) (−0.86) and interventricular septum thickness (IVSD) (−0.60) instead. Due to their interdependence, ECV and T1 relaxation time showed moderate significant correlation (*r* = 0.67). Moreover, moderate to weak significant negative relationships between LVEF and left ventricular global longitudinal strain (LV-GLS) (*r* = − 0.53), Creatinine (*r* = −0.20), and NT-proBNP (*r* = − 0.08) were found. ECV also showed moderate significant positive correlation with LVMMi (*r* = 0.68). Moreover, several echocardiographic parameters showed moderate to weak significant correlations with other echocardiographic parameters and with serum biomarkers. A correlation matrix showing correlation coefficients for all significant associations is presented in Fig. 4.

## Discussion

The present study on hybrid ^18^F-Florbetaben PET/MRI in CA is the first study to investigate prognostic imaging biomarkers derived from this modality in patients with various subtypes of CA. As a key finding, we identified the average RI from ^18^F-Florbetaben PET as the only independent and significant prognostic imaging biomarker of MACE free survival, which could not be observed for CMR and echocardiography parameters. This highlights the benefit of PET integration into diagnostic paradigms for assessment of CA.

First, we investigated the capacity of different imaging modalities to differentiate between CA subtypes. We could verify overall higher uptake values of AL-CA and better discrimination to other CA subtypes by established dynamic PET parameters, such as RI from 15 to 20 min, MTR and washout, outperforming echocardiography and CMR parameters (Table 2). Despite good available data for identification of CA, CMR and echocardiography are known to have limited utility in differentiation of the most common subtypes AL and wtATTR [24–27]. Especially echocardiography is falling short in differentiation of subtypes, whereas CMR based fibrosis markers, such as LGE pattern, increased T1 relaxation time and ECV have shown more extensive fibrotic changes in ATTR patients [27]. In this study, however, no CMR parameter showed differences between CA subtypes. In contrast to those imaging methods, amyloid specific PET have been shown to robustly differentiate between AL and ATTR subtypes [10–12, 28]. Our results confirm these evaluations which highlight the validity of ^18^F-Florbetaben PET in our patient cohort.

Next, we focused on the analysis of imaging biomarkers to predict MACE free survival. Whereas the other imaging parameters investigated, including PET RI from 15 to 20 min, were not prognostic, the newly introduced average RI was the only independent and significant prognostic imaging biomarker, that was capable to predict MACE free survival independent of the CA subtype. In difference to the diagnostic imaging parameters, we defined the average RI as a robust marker of cardiac amyloid burden displaying mean tracer retention over the entire dynamic uptake phase from 0 to 60 min.

Prognostic CMR parameters like native T1, ECV and LGE have been well-established as valuable biomarkers in cardiac diseases, including cardiac amyloidosis. T1 and ECV are particularly sensitive to myocardial fibrosis and interstitial expansion, which are hallmarks of amyloid infiltration, while LGE provides insights into the presence of scar tissue and myocardial damage [29, 30]. In CA, these CMR parameters have been shown to correlate with disease severity and prognosis, though their predictive value for clinical outcomes, such as MACE, remains somewhat variable, depending on disease subtype and imaging technique and remain relatively unspecific for cardiac amyloidosis [21, 31, 32].

In contrast molecular imaging biomarkers have only been scarcely investigated so farwith regards to outcome prediction in patients with CA [20, 33, 34]. Mostly, parameters were derived from bone scintigraphy [20, 35, 36]. For example, in a recent study by Caobelli et al., ^99m^Tc-DPD scintigraphy and quantitative SUVmax showed significant predication of MACE in a cohort of patients with ATTR-CA [33]. Moreover, Spielvogel et al. derived used an artificial intelligence-based approach to derive a quantitative cardiac amyloid marker from bone scintigraphy with different tracers that was prognostic for mortality [37]. In another recent study, Porcari et al. also identified diffuse right ventricular uptake in bone scintigraphy as an independent prognosticator of mortality [38].

Studies that evaluated PET images focused on AL-CA and identified visual cardiac uptake of ^11^C-Pittsburgh Compound B as prognosticator of 1-y mortality and quantitative left ventricular ^18^F-Florbetapir uptake as significant prognosticator of MACE [20, 36]. Interestingly, in comparison to a study by Clerc et al. who described a mediation of cardiomyocyte stress and heart failure (expressed by NT-proBNP) on the association of left ventricular ^18^F-Florbetapir percentage of injected dose and MACE in patients with AL amyloidosis, we did not reproduce a similar effect for RI in patients with different types of amyloidosis [20]. This may be explained by a different pathophysiology of the subtypes. Moreover, the average RI reflects a more comprehensive evaluation of cardiac amyloid burden than assessment of tracer uptake at a single imaging timepoint.

Advantages of the average RI lies in its applicability for different CA subtypes, whereas previous biomarkers were only defined for special CA subtypes. Whereas average RI is a time-integrated PET metric that reflects tracer binding and washout to amyloid fibrils, native T1 and ECV reflect tissue water relaxation that typically rises with interstitial expansion (e.g. fibrosis) in CA. Interestingly in our small mixed-subtype cohort, these metrics did not show positive correlation, which could be driven by differences in fibril composition/packing and tissue milieu at higher amyloid burdens (T1 plateau) where accessible binding sites (and thus RI) continue to increase. Residual technical variance (e.g. artificats) can further weaken or invert simple linear associations. Accordingly, we view these findings as indicator for convergent but non-redundant biological information rather than a contradiction of prior reports.

Thus, the hybrid PET/MR imaging approach allows not only valid diagnosis of CA and differentiation of CA subtypes but also outcome prediction. The added prognostic value of imaging derived biomarkers might be clinically implemented to optimize therapy management. Moreover, future evaluations might show if PET-based biomarkers can be used to monitor response to treatment of CA.

This is the first study that investigates hybrid PET/MR imaging in this setting, which allowed for a comprehensive correlation analysis between the echocardiographic, CMR, and PET parameters. No clear cross-modal correlation pattern between parameters was found, with strong association within PET parameters, but only scattered, pairwise cross-modal links and no shared prognostic signal for CMR/echo. Therefore, an integrated protocol for combination of PET and CMR can cover all relevant imaging parameters for analysis of function, tissue characterization, subtype differentiation and prognostic assessment. Because combined PET/MRI systems are not widely available, a sequential PET/CT followed by CMR workflow could be used as an alternative. Given that average RI was the only imaging biomarker associated with MACE in our cohort, we envisage a practical care pathway in which a high-risk average RI triggers additional or intensified follow-up (e.g. further diagnostic tests or eligibility for supportive devices).

Of major future interest are imaging methods for disease monitoring and response assessment to anti-amyloid treatments. Recently, ^99^Tc-DPD has shown decrease of tracer accumulation in patients with ATTR amyloidosis under treatment with Tafamidis and native T1 Mapping in CMR could assess therapy response in AL amyloidosis [39, 40]. However, the mechanism of bone scintigraphy tracers to amyloid is not fully understood and decrease in ^99^Tc-DPD uptake may not correlate with improvement of cardiac function. Here, hybrid PET/MRI could deliver a more comprehensive analysis and potentially fill the gap for disease monitoring and response to anti-amyloid treatments in both ATTR and AL subtypes as a “pan-amyloid-marker”.

##  Limitations

The present study has several limitations. Despite the prospective observational design and explorative nature of this study, recruitment of patients with cardiac amyloidosis who underwent ^18^F-Florbetaben PET/MRI proposes a major challenge, which is influenced by tracer logistics and long scan times. Therefore, the study included a limited number of patients overall and particularly small numbers in some amyloid subgroups, resulting in unbalanced group sizes. As a consequence, subtype-specific comparisons and multivariable models including several subtype categories (especially low number of AA-patients) were underpowered and prone to instability. To mitigate this, we primarily analysed the overall CA cohort and, where amyloid type was included in survival models, we pooled non-AL subtypes and used a binary classification (AL vs. non-AL). Accordingly, all subtype-specific findings should be interpreted as exploratory and hypothesis-generating. Additionally, given the small, mixed-subtype cohort, we intentionally did not derive a post-hoc composite CMR score to avoid overfitting and are cautious about validity of absent prognostic value of CMR parameters. Therefore, future prospective evaluations are necessary to validate the results and estimate the clinical benefit.

## Conclusion

Our results ^18^F-Florbetaben support current data on detection and imaged-based discrimination of CA subtypes. Moreover, we introduced the average RI as novel independent prognostic imaging biomarker, that was independent of the CA subtype. Further studies are now needed to investigate these potential predictive markers with respect to overall outcome and to establish statistically significant cut-offs to identify at-risk patients. Future work should investigate average RI cut-points in a prospective management pathway to determine impact on clinical management and reduce MACE and improve patient-centered outcomes.

## Supplementary Information

Below is the link to the electronic supplementary material.

Supplementary Material 1

Supplementary Material 2

## Abbreviations

AA: Serum-Amyloid A
AL: Amyloid ligh tchain
(w/ht) ATTR: (wildtype/hereditary) Transthyretin
CA: Cardiac Amyloidosis
CMR: Cardiac magnetic resonance
ECV: Extracellular volume
MACE: Major adverse cardiac events
RI: Retention index
