# Supplementary material for: Prognostic parameters and detection of cardiac amyloidosis with hybrid 18F-Florbetaben-PET/MRI: an exploratory observational study
Source: Eur J Nucl Med Mol Imaging. 2026 Feb 7;53(6):3973–84. doi: 10.1007/s00259-025-07733-x (PMC13121523; doi:10.1007/s00259-025-07733-x)
Supplement: Supplementary file 2 — Supplementary Material 2 [file 259_2025_7733_MOESM2_ESM.docx]

**Supplemental Material**

**PET Acquisition and Reconstruction**

Cardiac PET scans comprised 1 bed position. PET image reconstruction was performed using 3D ordinary Poisson ordered subset expectation maximization with 3 iterations and 21 subsets, applying a 344 matrix (2 × 2 × 2 mm voxel size) and a Gaussian filter with 5.0 mm full width at half-maximum. For attenuation correction of PET data, a four-compartment model attenuation map was calculated from fat-only and water-only Dixon-based MRI sequences by segmentation into background, lung, fat, and soft tissue.

PET list-mode data were reconstructed into the following time frames: 12 frames with 5 s each, 6 frames with 10 s each, 4 frames with 30 s each, 6 frames with 60 s each, 8 frames with 300 s and 1 frame of 600 s (3600 s = 60 min in total). Additionally, for visual inspection, static images from PET data acquired at 40-60 min after tracer injection were reconstructed.

**CMR Protocol**

CMR scans were perfomred using a clinical standard protocol as previously described (Papathansiou et. al, 2018). After localizers and cine imaging with True Fast Imaging with Steady-State Free Precession (trueFISP) sequence), T1 mapping, basal and midventricular short-axis and 4-chamber long-axis images were acquired by the modified Look-Locker inversion recovery (MOLLI). After the standard LGE imaging the post contrast T1 measurement was repeated approx. 20 min p.i. with the MOLLI sequence. T1 measurement was performed by drawing a region of interest in the basal to mid-septum of the appropriate 4-chamber map. For extracellular volume (ECV) measurement, a single region of interest was drawn in each of the 4 required areas: myocardial T1 estimates (basal to mid-septum in 4-chamber map) and blood T1 estimates (left ventricular cavity blood pool in 4-chamber map) before and after contrast administration. Hematocrit was available in all subjects. ECV was calculated according to the established formula:

myocardial ECV = (1−hematocrit) (Δ*R*1_myocardium_/Δ*R*1_blood_), where *R*1 = 1/*T*1.

**Kinetic parameters calculation**

**Retention index from 15 to 20 min**

The 18F-florbetaben myocardial RI was computed as the mean LV myocardial tissue radiotracer concentration between 15 and 20 min after injection of 18F-florbetaben, divided by the integral of the blood pool 18F-florbetaben time–activity curve from 0 to 17.5 min after injection (Law et al.).

**Average retention index f**

The 18F-florbetaben myocardial RI at each imaging timepoint was computed according to the definition by Genovesi et al. Afterwards we calculated the mean of the RI from each frame for a more robust analysis of the amyloid burden over the entire uptake curve.

**MTR**

Percentage myocardial tracer retention (MTR, given in [%]) was calculated as the change in LV myocardial SUVmean on summed-framed images of the first 5 min after i.v. injection of the tracer and between 40 and 60 min (adapted from Kircher et. al).

**Washout**

The tracer washout rate was calculated as follows:

$$Washout=\left( \frac{\left( SUVmean 5\min- SUVmean 60min \right)}{SUVmean 5min} \right)x 100\%$$

**Statistics**

Continuous variables are presented as mean ± standard deviation or median (interquartile range), as appropriate, and categorical variables as counts (percentages). Distribution of continuous variables was assessed using the Shapiro–Wilk test. For comparisons of continuous variables between cardiac amyloidosis (CA) subtypes, we used the Kruskal–Wallis test. Associations between categorical variables were evaluated using the χ² test or Fisher’s exact test, as appropriate.

The primary endpoint was MACE-free survival, defined as the time from the index PET/MRI examination to the first occurrence of a major adverse cardiovascular event (MACE). Time-to-event was calculated in days as the difference between the examination date and the date of the first MACE or last clinical follow-up. Patients without MACE at last contact and those lost to follow-up were censored at the date of last available clinical information. The event indicator (censor_Mace) was coded as 1 for occurrence of MACE and 0 for censored observations.

Univariable associations between individual clinical, biomarker, echocardiographic, and PET/MR parameters and MACE-free survival were first examined using Cox proportional hazards regression models with each predictor entered separately as a continuous variable. In a secondary step, continuous predictors were dichotomized at the sample median to facilitate clinical interpretation and graphical display. For these dichotomized variables, the lower-risk group (below or equal to the median, or clinically favourable category) was used as the reference category. For descriptive survival analyses, Kaplan–Meier curves were generated for selected dichotomized variables, and groups were compared using the log-rank test. Median MACE-free survival with 95% confidence intervals (CI) was estimated from Kaplan–Meier curves. Hazard ratios (HR) with 95% CI were reported for all Cox models. Multivariable Cox models were constructed including clinically relevant variables and those with evidence of association in univariable.

To explore the interrelationships between imaging and biomarker parameters, we calculated pairwise Pearson correlation coefficients using all available observations (complete-case analysis). Correlation matrices were visualized using hierarchical clustering and significance testing with corresponding P values.

We additionally performed causal mediation analyses to investigate whether the association between average RI and MACE-free survival was mediated by NT-proBNP and high-sensitivity troponin (hs-Tn). For these analyses average RI, NT-proBNP, and hs-Tn were log-transformed to reduce skewness. The mediator was modelled using linear regression with average RI as the exposure. MACE-free survival was modelled using a parametric accelerated failure time model with a log-normal distribution. The average causal mediation effect (indirect effect via the mediator) and the average direct effect of average RI were estimated using the mediation package in R, with uncertainty quantified by non-parametric bootstrap with 1,000 simulations and robust standard errors.

Due to the exploratory nature of this study, no formal adjustment for multiple testing was applied; P values are therefore descriptive. All tests were two-sided and P values < 0.05 were considered statistically significant. Analyses were performed using GraphPad Prism (Version 10.2.3 for Windows, GraphPad Software, Boston, MA, USA) and R statistical software (Version 4.4.1, R Foundation for Statistical Computing, Vienna, Austria), including the packages survival, survminer, gtsummary, corrplot, and mediation.

**Supplemental Tabl. 1.**

| **Type of MACE** | **Total = 14** |
| --- | --- |
| Hospitalization due to heart failure | 9 (64.3%) |
| Myocardial Infarction / CAD & Coronary stenting | 3 (21.4%) |
| Arrhythmia / Cardioverter implantation | 1 (7.1%) |
| Death | 1 (7.1%) |

**Supplemental Tabl. 2.**

| **Subtype (No. MACE)** | **Median days to MACE** | **Mean (SD) days to MACE** |
| --- | --- | --- |
| Overall (N = 14) | 298 | 411 (325) |
| AL (N = 4) | 452 | 509 (385) |
| wtATTR (N = 7) | 226 | 349 (372) |
| hATTR (N = 1) | 578 | 578 (0) |
| AA (N = 2) | 350 | 350 (45) |

**Supplemental Fig. 1**

**
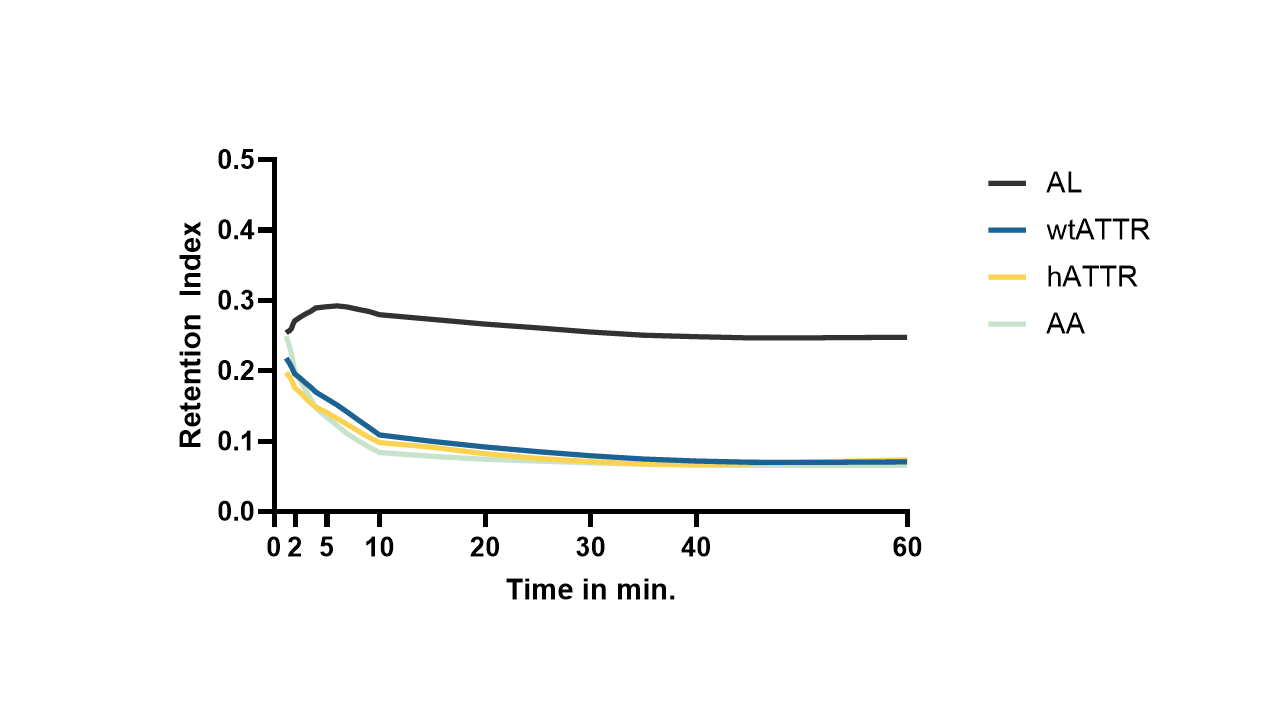
**

**Suppl. Fig. 1. Trend of mean retention index for amyloidosis subtypes.**
